# Mushroom-Based Supplements in Italy: Let’s Open Pandora’s Box

**DOI:** 10.3390/nu15030776

**Published:** 2023-02-02

**Authors:** Samuele Risoli, Cristina Nali, Sabrina Sarrocco, Arrigo Francesco Giuseppe Cicero, Alessandro Colletti, Filippo Bosco, Giuseppe Venturella, Agata Gadaleta, Maria Letizia Gargano, Ilaria Marcotuli

**Affiliations:** 1 Department of Agriculture, Food and Environment, University of Pisa, Via del Borghetto 80, 56124 Pisa, Italy; 2 Nutrafood Research Center, University of Pisa, Via del Borghetto 50, 56124 Pisa, Italy; 3 Medical and Surgical Sciences Department, University of Bologna, 40126 Bologna, Italy; 4 IRCCS AOU S. Orsola di Bologna, 40126 Bologna, Italy; 5 Department of Drug Science and Technology, University of Torino, 10124 Torino, Italy; 6 U.O. Anesthesia and Intensive Care MiSC, AOUP Complementary Medicine Oncology Integrated, University Hospital Trust of Pisa, 56126 Pisa, Italy; 7 Department of Agricultural, Food and Forest Sciences, University of Palermo, Viale delle Scienze, Bldg. 5, 90128 Palermo, Italy; 8 Department of Soil, Plant, and Food Sciences, University of Bari, Via G. Amendola, 165/A, 70126 Bari, Italy

**Keywords:** nutraceuticals, mushrooms, nutritional supplements, reishi, shiitake, *Agaricus blazei*, functional foods, quality

## Abstract

Mushrooms and derivates are well known to the scientific community for having different health benefits and exhibit a wide range of pharmacological activities, including lipid-lowering, antihypertensive, antidiabetic, antimicrobic, antiallergic, anti-inflammatory, anticancer, immunomodulating, neuroprotective and osteoprotective actions. In Europe, medical mushrooms are mainly marketed in the form of food supplements as single components or combined with other nutraceuticals. In this context, the first peculiarity that distinguishes it is the safety established through the “history of consumption” that characterizes that mushroom. However, the cultivation of medicinal mushrooms on a large scale is performed mainly in China, where most of the production facilities do not have internationally recognized good manufacturing practices, despite that many European companies that sell myotherapies are supplied by Chinese manufacturers. This is particularly evident in Italy, where an arsenal of mushroom products is marketed in the form of powders and extracts not always of ascertained origin and sometimes of doubtful taxonomic identification, and thus not meeting the quality criteria required. The growing interest in mycotherapy involves a strong commitment from the scientific community to propose supplements of safe origin and genetic purity as well as to promote clinical trials to evaluate its real effects on humans. The purpose of this research is to analyze different mushroom-based dietary supplements used in medicine as monotherapy on the Italian market and to evaluate their composition and quality. The molecular identification of the sequences with those deposited in GenBank allowed for identifying 6 out of 19 samples, matching with those deposited belonging to the species indicated in the label, i.e., *Lentinula edodes* (samples 1, 4, 12 and 18) and *Ganoderma lucidum* (samples 5 and 10). Samples containing Ganoderma, labeled in the commercial product as *G. lucidum*, showed sequences that showed homology of 100% and 99% with *G. resinaceum* and *G. sichuanense*. An additional investigation was carried out in order to determine the active fungal ingredients, such as ergosterol, aflatoxins, heavy metals, nicotine and total glucan. The results obtained and shown in the manuscript highlight how the data were not only in line with what is expected with respect to what is indicated in the labels.

## 1. Introduction

Mushrooms are appreciated for their culinary and nutritional value and are increasingly valued for their medicinal properties, especially for their activities on cardiometabolic parameters, the immune system, and as anti-inflammatory and anticancer agents [1].

The use of medicinal mushrooms in traditional Chinese medicine (TCM) dates back 3000–7000 years [2]. The first cultivation of medicinal mushrooms (*Lentinula edodes* (Berk.) Pegler), shiitake, dates back to 600–1000 BC [3]. The Shénnóng Běncǎo Jīng, attributed to Emperor Shennong (2800 BC), reported a number of drugs harmless to humans with “stimulating properties”, including the reishi mushroom (*Ganoderma lucidum* (Curtis) P. Karst) [4].

Medicinal mushrooms have been supposed to be potentially useful for the prevention and treatment of different diseases in humans, farms and domestic animals [5]. Mushrooms are used not only as dietary food (functional foods) but also in the form of dietary supplements, nutraceuticals and mushroom-based supplements [6]. The polysaccharides and polysaccharide–protein complexes are abundant in medicinal mushrooms, and β-glucans are mostly used because of their immunomodulating activities in adjunct tumor therapy [7]. Additionally, β-glucans have a significant impact on the health of microbial flora in the human gut and have been recognized as potential pharmaceutical preventative agents of diet-related chronic diseases when absorbed at appropriate doses [8]. A regular fiber diet can reduce serum cholesterol and glucose levels and thus the risk of obesity, type II diabetes and cardiovascular disease [9].

In addition, there is an increasing interest in the medicinal use of mushroom-derived nutraceuticals, especially that from the *Basidiomycetes* family, such as *Agaricus blazei* Murill, *G*. *lucidum, Hericium erinaceus* (Bull.) Pers. and *Grifola frondosa* (Dicks.) Gray has been reported to exhibit anti-inflammatory and immune-modulating activities due to the presence of bioactive molecules, including antibiotics, for example, penicillin and griseofulvin, and chemically highly diversified anti-inflammatory compounds, such as polysaccharides, terpenoids, phenolic compounds, glycerides and other low molecular weight molecules [10]. For this reason, the great richness of the mycocomplex could explain the great versatility of therapeutic action attributed to medicinal mushrooms, especially in this pandemic period [11]. In fact, several in vitro studies have shown the ability of medicinal mushrooms to inhibit different virus proteases and reduce the “cytokine storm”, suggesting its potential activity against the main proteases of coronaviruses [12]. In addition, several randomized clinical trials highlight the ability to stimulate both innate and acquired immunity-modulating NK cells, macrophages and T cells, and against chemotherapeutic myelosuppression, it is also one of the most serious deleterious effects of chemotherapy [13,14].

The cultivation of medicinal mushrooms on a large scale is performed mainly in China, where the mushroom-based nutraceutical and pharmaceutical products industry is highly developed [15]. It should be noted that most of the production facilities in China do not have internationally recognized good manufacturing practices (GMP) and that many European companies that sell mushroom-based products are supplied by Chinese manufacturers. In particular, products purchased from abroad in the form of powders and extracts are not always of ascertained origin and sometimes of doubtful taxonomic identification [16].

This is particularly evident in Italy, where products derived from medicinal mushrooms placed on the market often do not meet the required quality criteria. In addition, the cultivation of medicinal mushrooms in Italy remains at present underdeveloped or even non-existent, so it is difficult to ascertain whether such cultivations really exist and if they do, where they are located, and what is the origin of the biological material and substrate used for cultivation [17]?

The growing interest in mycotherapy requires a strong commitment from the scientific community to propose supplements of safe origin and genetic purity and to expand clinical trials to evaluate their real effects on humans.

For the above-mentioned reasons, the purpose of this research is to analyze different mushroom-based supplements available on the Italian market and to evaluate their composition.

## 2. Materials and Methods

### 2.1. Mushroom-Based Supplements Samples

In order to analyze the composition of some of the main mushroom-based supplements actually available on the Italian market and regularly used for their medicinal properties, 19 samples, marketed by 6 different companies, were submitted to molecular and biochemical analysis aimed to (i) identify the mushroom species used to extract the bioactive ingredient; (ii) quantify the ergosterol (ERG) and total glucans content; and (iii) check the possible presence of hazardous compounds, such as mycotoxins and heavy metals, where these last are due to subsequent contaminations occurring during the production/packaging processes. The samples consisted of dietary supplements used in medicine in the form of encapsulated powder and were used as they were, without any alteration, and not expired. In Table 1, a list of the 19 samples and the corresponding mushroom species used to extract polysaccharides (as indicated on the label of the commercial product) is reported. Information about the mushroom-based supplements products and the name of the 6 companies will be available to readers upon request from the corresponding authors.

### 2.2. Molecular Identification of Fungal Active Ingredients

Molecular identification of the fungal isolates used as a source of glucans for mushroom-based supplements was performed by sequencing the internal transcribed spacers (ITS). Genomic DNA was extracted from 100 mg of each sample (from the powder inside three capsules or by powdering three capsules from the same batch, depending on the format of the samples) by using the Quick-DNA Fungal/Bacterial kit (Zymo Research, Irvine, CA, USA), according to the manufacturer instructions. Complete ITS 1 and 2 sequences, including the 5.8S gene of the nuclear ribosomal DNA, were amplified according to Sarrocco et al. [18] by using the universal primers ITS5 and ITS4 [19]. Amplicons were purified by the QIAquick PCR Purification Kit (Qiagen, Maryland, USA), while their sequencing was performed by Bio Molecular Research (BMR, Padova, Italy).

Resulting sequences were blasted in the NCBI Genome Database (GenBank, https://www.ncbi.nlm.nih.gov/genbank/, accessed on 1 December 2022) in order to identify, at least at the genus level, the mushrooms included in the analyzed products, according to the percentage of homology with deposited sequences.

### 2.3. Determination of Ergosterol

For each sample, a total of 3 independent replicates (capsules) were analyzed. Each capsule was finely ground before the extraction step. Ergosterol was determined according to Bååth and Anderson [20] with minor modifications. An amount of 250 µL cyclohexane and 1000 µL of 10% KOH (in methanol, MeOH) were added to 0.25 g of product powder. After 15 min of ultrasonic treatment, tubes were put in a 70 °C water bath for 90 min. Following this phase, 250 µL of distilled water and 500 µL of cyclohexane were added, the tubes were firstly vortexed for 30 s, centrifuged, and the top phase was removed. The residual solution was rinsed once again with 500 µL of cyclohexane before the mixed cyclohexane fraction was evaporated at room temperature. Before measuring the amount of ERG, samples were dissolved in 500 µL of the MeOH by heating at 40 °C for 15 min and then filtered through a 0.45 µm filter. The separation was performed for 20 min at 30 °C by an ultra-high pressure liquid chromatography (UHPLC) Dionex UltiMate 3000 system (Thermo Scientific, Waltham, MA, USA) equipped with an Acclaim 120 C18 column (5 μm particle size, 4.6 mm internal diameter × 150 mm length; Thermo Scientific, Waltham, MA, USA), and a Dionex Uvd340U Uv/Vis Detector (Thermo Scientific, Waltham, MA, USA) using UV light at 280 nm. The mobile phase was MeOH 100%, and the flow rate was 0.8 mL min^−1^. To quantify the ERG content, known amounts of pure standard (0.1–100 ng mL^−1^) were injected into the UHPLC system, and an equation correlating peak area to ERG concentration was formulated (results are reported as µg g^−1^ DW).

### 2.4. Determination of Aflatoxins

Regarding aflatoxins (AFTs), extraction was performed according to Roch et al. [21] with minor modifications. An amount of 0.25 g of a finely ground matrix was added with 1 mL of methanolic solution (60:40 in UHPLC grade) and vigorously vortexed for 3 min. Subsequently, the samples were centrifuged for 10 min at 12,000× *g* at room temperature, and 400 µL of supernatant was withdrawn and diluted with 1600 µL of phosphate buffer saline (PBS 0.05 M, pH 7.4). The diluted was filtered and purified by immune-affinity columns (IAC, AFLA B&G SELECT-OR, OR SELL, S.p.a., Modena, Italy) in accordance with the method provided by the manufacturer, evaporated and successfully re-suspended in H_2_O:MeOH (55:45 *v*/*v*, UHPLC grade water). The separation was performed for 30 min at 30 °C by a UHPLC Dionex UltiMate 3000 system (Thermo Scientific, Waltham, MA, USA) equipped with a ZORBAX Eclipse Plus C18 column (5 μm particle size, 4.6 mm internal diameter × 150 mm length, Agilent Technologies, Santa Clara, CA, USA), and an UltiMate™ 3000 Fluorescence Detector (Thermo Scientific, Waltham, MA, USA) with excitation at 362 nm and emission at 420 nm after post-column derivatization through a UVE™ Photochemical Reactor for Aflatoxin Analysis (254 nm lamp; 240 VAC, 50/60 Hz, LCTech, Obertaufkirchen, Germany). The flow rate was 1 mL min^−1^, and the mobile phase was H_2_O:MeOH (55:45 *v*/*v*, UHPLC grade water). To quantify the AFTs content, known amounts of pure mixed standards (0.1–100 ng mL^−1^ Aflatoxin Mix 4 solution, Romer Lab, Getzersdorf, Austria) were injected into the UHPLC system. In order to quantify AFTs concentration (reported as the sum of the four major aflatoxins types (B1, B2, G1 and G2 AFTs) in µg kg^−1^ DW), an equation correlating peak area was formulated.

### 2.5. Determination of Transition and Heavy Metals

The transition metals (iron, both ferric (Fe^3+^) and ferrous (Fe^2+^); copper (Cu); cobalt (Co); manganese (Mn); nickel (Ni); and zinc (Zn)) detection through ion chromatography (IC) in this study was optimized from the example application “Determination of trace level of transition metals using CS5A 2-mm column and preconcentration” presented in the CS5A manual, which was the separator column used in this study. For the quantification of transition metals, around 0.5 g of finely ground powder from each sample was digested (100 °C, 1 h) in 4 mL of 50% HNO_3_ diluted with 1 mL 30% H_2_O_2_ and successively filtered through a 0.22 µm filter. The separation was performed for 20 min at room temperature by an IC Dionex Aquion system (Dionex Aquion, 2-mm IonPac CG5A Guard and 2-mm CS5A Analytical Column; Thermo Scientific, Waltham, MA, USA) and a Dionex VWD UV/Vis Detector (Thermo Scientific, Waltham, MA, USA). An amount of 50 µL of the sample was eluted with Dionex MetPac PDCA eluent (Thermo Scientific, Waltham, MA, USA, flow rate 1.2 mL mL^−1^) and 0.5 mM PAR (Dionex MetPac PAR, Thermo Scientific, Waltham, MA, USA; flow rate 0.7 mL mL^−1^) as a post-column diluent for the derivatization. Detection was performed using UV light at 530 nm, transition metals standards were always dissolved in dilute acid solutions and can also be used as IC standards (0.1–100 mg mL^−1^) and an equation, correlating peak area to metals concentration, was formulated. The quantification of arsenic (As), cadmium (Cd), mercury (Hg) and chromium (Cr) was carried out starting from 25 g of the sample through an inductively coupled plasma mass spectrometry (ICP-MS) system, in accordance with the current European legislation (UNI EN ISO 17294-2:2016).

### 2.6. Determination of Nicotine

For nicotine extraction, this study referred to the method described by Kang et al. [22] in accordance with (EURL, 2016). An amount of 0.5 g of dried sample was placed in a 50 mL tube and added with 15 mL of distilled water. After vortexing, 5 M NaOH was used to correct the pH of the extract to be in the 10–11 range. Successively, 6 g of MgSO_4_ and 1.5 g of NaCl were added, the mixture was shaken for 15 min and then centrifuged for 5 min (1590× *g*), and the supernatant was filtered through a 0.45 μm membrane filter (Sartorius Minisart^®^, Goettingen, Germany). Nicotine quantification was carried out using an Agilent 8890B GC-MS System equipped with a 5977B single quadrupole mass detector (Agilent Technologies, Inc., Santa Clara, CA, USA). An Agilent DB-5 MS column C (30 m × 0.25 mm × 0.25 μm; Agilent Technologies, Inc., Santa Clara, CA, USA) was used for analysis. A total amount of 2 μL of the sample was injected in splitless injection mode (235 °C for evaporation, Helium carrier gas, flow rate of 1 mL min^−1^). Initially, the oven temperature was set to 70 °C and maintained for 1 min, and then the temperature was raised to 150 °C (25 °C min^−1^), 210 °C (12 °C min^−1^) and 280 °C (25 °C min^−1^). The post-run temperature was maintained at 310 °C (3 min). In all the analyzed samples, nicotine content results are always under the instrumental detection limit.

### 2.7. Total Glucan Quantification in Mushroom-Based Supplements Products

Total glucan was measured using controlled acid hydrolysis with H_2_SO_4_. Specifically, the glucose released was measured using glucose oxidase/peroxidase and GOPOD reagent, following the instruction of the commercial kit from Megazyme [23]. The measurement was carried out on 5 different capsules, considered biological replicates, each of which was tested in duplicate, and on the mixture of 5 single capsules in duplicate to estimate differences between the two methods.

## 3. Results

### 3.1. Molecular Identification of Fungal Active Ingredients

When gDNA extracted from the capsules analyzed was used to amplify ITS sequence, all the PCR resulted in an amplicon of around 600–700 bp length, but the DNA of samples 6 and 19 was degraded, and the amplification of sample 13 resulted in several bands of different length belonging to yeasts species (such as *Aureobasidium* spp., *Geotrichum* spp. or *Teunomyces* spp.).

Comparison of the sequences with those deposited in GenBank allowed for identifying 15 out of 19 samples, with percentages of homology generally higher than 98%, but for samples 5, 14 and 17, the percentages of homology with reference sequences were 88%, 93% and 92%, respectively.

In Table 2, for each sample, the identification, percentage of homology with deposited sequences, and their accession number are listed. In detail, only for samples 1, 4, 10, 12 and 18 did the amplified sequences match with those deposited belonging to the species indicated in the label, i.e., *Lentinula edodes* (samples 1, 4, 12 and 18) and *Ganoderma lucidum* (samples 10), while for sample 5, the 88% of homology with *G. lucidum* can not be considered reliable for a secure identification at the species level. With respect to the other products containing *Ganoderma*, while on the label of the commercial product *G. lucidum* was indicated, identification of submitted sequences showed 100% and 99% of homology with *G. resinaceum* for samples 2 and 16, respectively, while the sequence obtained for sample 14 showed a 93% of homology with that of *G. sichuanense*.

A higher mismatch was obtained for the commercial products, whose bioactive ingredients should have been extracted from *Agaricus blazei*. Samples 3, 9, 11 and 17 showed a 98% of homology with *G. resinaceum*, 99% with *Grifola frondosa*, 99% with *A. subrugescens* and 92% with *Cordyceps militaris*, respectively. Finally, both samples 7 and 8, whose active ingredients should have been *L. edodes* and *G. lucidum*, respectively, gave rise to ITS sequences showing 99% (sample 7) and 100% (sample 8) of homology with *G. frondosa*.

### 3.2. Determination of Ergosterol

The contents of ERG (expressed as µg g^−1^ DW, determined for each sample on three different capsules) for the sample are shown in Table 3. In batch A, the highest concentration of ERG was measured in product 2 (561.60 µg g^−1^), while in products 1 and 3, the ERG contents were, respectively, 452.85 and 151.93 µg g^−1^. In batch B, product 5 was found to be the one with higher quantities of ERG (332.58 µg g^−1^) in comparison to the contents recorded in products 4 and 6 (34.38 and 43.6 µg g^−1^, respectively). In batch C, the highest amount of ERG was registered for product 7 (129.91 µg g^−1^), while in products 8 and 9, the ERG contents were 31.32 and 40.75 µg g^−1^, respectively. In batch D, minor quantities of ERG were found, and product 11 was found to be the one with a higher content (20.19 µg g^−1^) compared to the values recorded in products 10 and 12 (15.87 and 3.45 µg g^−1^, respectively). In batch E, the highest value was recorded in product 14 with 132.20 µg g^−1^ in comparison with those recorded in the other two samples (1.79 and 21.53 µg g^−1^, respectively, products 13 and 15). Moreover, in the last batch (batch E), product 17 turned out to be the one with the highest concentrations (70.29 µg g^−1^) if compared to the values recorded for products 16 and 18 (44.58 and 52.14 µg g^−1^, respectively). In sample 19, the ERG concentration was 8.54 µg g^−1^. Analysis of the variance of ERG content is shown in Table 4 and highlights the variability both between and within the groups (sample) for ERG contents.

### 3.3. Determination of Aflatoxins

Regarding AFTs analysis (expressed as µg g^−1^ DW, determined for each sample on three different capsules), traces of mycotoxins were only found in samples belonging to batches A, B and C (Table 5). In the first batch (batch A), the highest amount of AFTs was recorded in product 2 (2.68 µg kg^−1^), while in products 1 and 3, the AFTs contents were, respectively, 0.12 and 1.21 µg kg^−1^, while in batch B, the highest content of AFTs was measured in product 5 (3.16 µg kg^−1^) if compared with product 4 (0.17 µg kg^−1^); in sample 6, AFTs was non detected. In batch C, the highest values of AFTs were recorded, with concentrations of 2.62, 1.72 and 4.99 µg kg^−1^ (sample 7, 8 and 9, respectively) of the total amount of AFTs; only in sample 9, the concentration of AFTs results higher than that allowed by Commission Regulation (EC) No 1881/2006 for maximum levels for total AFTs contamination in foodstuffs. Analysis of the variance of AFTs contents is shown in Table 6.

### 3.4. Determination of Transition and Heavy Metals

Total iron content (Fe^2+^ and Fe^3+^), expressed as µg g^−1^ DW, was determined for each product on three different capsules (Table 7). The results of 19 mushroom-based supplements showed variability in total iron contents in different samples and between samples from the same batch. In detail, the values ranged from 2.041 (sample 12) to 110.803 µg g^−1^ (sample 9), with an overall mean value of 29.87 µg g^−1^. Among batch A, the highest concentration of total iron content was measured in sample 1 (62.36 µg g^−1^), while in samples 2 and 3, the total iron contents were, respectively, 19.66 and 8.24 µg g^−1^. Among batch B, product 6 was found to be the one with higher quantities of total iron (104.02 µg g^−1^) in comparison to the contents recorded in samples 4 and 5 (26.87 and 23.64 µg g^−1^, respectively). In batch C, the highest amount of total iron was registered in sample 9 (110.80 µg g^−1^), while in samples 7 and 8, the total iron contents were 8.86 and 62.29 µg g^−1^, respectively. In batch D, minor quantities of total iron were found in sample 12 (2.04 µg g^−1^), while in samples 10 and 11, the total iron contents were, respectively, 2.91 and 6.69 µg g^−1^. Among batch E, sample 13 turned out to be the one with the highest concentrations (7.51 µg g^−1^) if compared to the values recorded for samples 14 and 15 (4.72 and 6.12 µg g^−1^, respectively). Among the last batch (batch F), the highest value was recorded in sample 17, with 27.29 µg g^−1^ in comparison with those recorded in the other two samples (15.03 and 13.34 µg g^−1^ in samples 16 and 18, respectively). In sample 19, the total iron content was 55.00 µg g^−1^. Considering the total iron content obtained using the mixture from three different capsules, the ANOVA analysis (Table 8) underlined how the variation in total iron content between capsules was statistically different (*p* < 0.001), and a single capsule from the same batch did not have the same total iron amount. Within the samples analyzed, concentrations of other transition metals analyzed (copper, cobalt, manganese, nickel and zinc) are always under the instrumental detection limit, while the concentration of As, Cd, Hg and Pb amounts are always lower than the European legal limits (Commission Regulation (EC) No 1881/2006 of 19 December 2006), as reported in Table 7.

### 3.5. Glucan Content in Mushroom-Based Supplements

Total glucan content, expressed as a percentage of grams of glucan on 100 g of products, was determined primarily for each product on five different capsules (Table 8 and Table 9). The results of 19 mushroom-based supplements showed variability in glucan contents in different species and between capsules from the same batch. In detail, the values ranged from 19.15 (sample 8) to 60.05 g or 100 g^−1^ (sample 13), with an overall mean value of 38.71 g 100 g^−1^. Considering the glucan content obtained using the mixture from five different capsules and compared to the mean of the values derived from single capsule analysis, the values were slightly different for some mushroom-based supplements products, such as sample 2, while for some other the differences were statistically significant (e.g., sample 2, 3 and 12) as shown in Table 10. Analysis of ANOVA underlined how the variation in glucan content between capsules was statistically different (*P* < 0.001), and a single capsule from the same batch had not the same glucan amount.

## 4. Discussion

Nutraceutical is a syncretic neologism from “nutrition” and “pharmaceuticals” coined by dr. Stephen de Felice in the late 1980s. It is the discipline that studies enriched foods, functional foods and food supplements (including botanicals and mushrooms), which may have a preventive or, in some cases, a therapeutic role on one or more pathologies or risk factors [24]. However, what clinicians define nutraceuticals by law is a dietary supplement, which falls within the sectoral legislation (Directive 2002/46/EC) as: “*food products intended for the supplementation of the common diet and which constitute a concentrated source of nutrients, such as vitamins and minerals or other substances, having a nutritional or physiological effect, in particular, but not exclusively, amino acids, essential fatty acids, fibers and extracts of vegetable origin, both as single and multi-compounds, commercialized in pre-dosed forms*”. Thus, the dietary supplement is considered a food by the regulatory authority; as such, the first characteristic that distinguishes it is the safety established through the history of consumption that characterizes that particular substance. However, although the food supplement is typically considered a “natural product” with the meaning of the total safety of itself, the scientific literature is not exempt from reports of adverse effects caused by nutraceuticals, especially in frail or pluri-pathological patients [25]. In particular, some adverse events can be attributable to the presence of unwanted contaminants: for example, it was reported that citrinin induced hepatotoxicity, a mycotoxin contained in fermented red yeast rice [26]. In addition, the non-obligation of both in vitro and in vivo testing of nutraceuticals represents the greatest limitation of this category of molecules and, as a direct consequence, it is now possible to find on the market products of all kinds, with extremely heterogeneous substances, in various combinations and dosages, and in extremely different pharmaceutical forms. This is particularly true for the world of medicinal mushrooms. Despite the fact that randomized controlled trials conducted to date highlight a good safety profile for nutraceutical mushrooms, many of these have not been evaluated for their safe human use using modern analytical approaches, and some toxicological endpoints may be opaquer. In fact, obtaining safety data for developmental and reproductive toxicity, genotoxicity, and chronic endpoints can prove particularly difficult. Complicating the evaluation of such fungi, modern cultivation practices, and preparations are rarely consistent with traditional medicinal uses. While fruiting bodies are most often the portion of the organism used in TCM [27], commercial raw materials typically consist of the fungi’s mycelium, which grows more quickly and is, therefore, less expensive to produce. Moreover, the geographical place of cultivation and the growing conditions could influence the secondary metabolite profile of fungi, including the presence of contaminants such as mycotoxins that negatively impact health [28]. To date, a multifaced approach is available in order to assess the safety of fungi as dietary supplements. This approach should include a critical starting review of the scientific literature for that specific fungal species, which may be confirmed through a genetic analysis (DNA identification) [29]. In addition, an analysis of the fungal toxins should be encouraged, starting from a database of known fungal metabolites. In this regard, the authors highlighted the higher mismatch obtained through the genetic analysis between the label of the commercial product *G. lucidum* indicated and the identification of submitted sequences that showed 100% and 99% of homology with *G. resinaceum* (for samples 2 and 16, respectively) and a 93% of homology with *G. sichuanense* (for sample 14). Similar results were obtained for the commercial products based on *Agaricus blazei*. Samples 3, 9, 11 and 17 showed a 98% of homology with *G. resinaceum*, 99% with *Grifola frondosa*, 99% with *A. subrugescens* and 92% with *Cordyceps militaris*, respectively. Finally, both samples 7 and 8, whose active ingredients should have been *L. edodes* and *G. lucidum*, respectively, gave rise to ITS sequences showing 99% (sample 7) and 100% (sample 8) of homology with *G. frondose*.

Furthermore, the AFTs analysis showed traces of mycotoxins. In the first batch (batch A), the highest amount of AFTs was recorded in sample 2 (2.68 µg kg^−1^), while in samples 1 and 3, the AFTs contents were, respectively, 0.12 and 1.21 µg kg^−1^, while in batch B, the highest content of AFTs was measured in sample 5 (3.16 µg kg^−1^) if compared with product 4 (0.17 µg kg^−1^); in sample 6, AFTs was non detected. In batch C, the highest values of AFTs were recorded, with concentrations of 2.62, 1.72 and 4.99 µg kg^−1^ (product 7, 8 and 9, respectively) of the total amount of AFTs; more troubling is product 9, in which the concentration of AFTs results higher than that allowed by Commission Regulation (EC) No 1881/2006 for maximum levels for total AFTs contamination in foodstuffs. All of this is particularly relevant since the dosages of mushrooms demonstrated to be effective on human health parameters are high, and the effectiveness has been mainly observed for middle-long term exposition so that the highest safety profiles should be warranted while the detected concentrations of heavy metals and nicotine do not seem to represent a problem.

Very important are also the results of 19 mushroom-based supplements on glucan contents which displayed variability in different species and between capsules from the same batch. In detail, the values ranged from 19.15 (sample 8) to 60.05 g 100 g^−1^ (sample 13), with an overall mean value of 38.71 g 100g^−1^. The results obtained from the analysis of the glucan content confirm the lack of uniformity within the batches, as already observed from the ERG (a good indicator of fungal biomass) analysis. Moreover, in this case, indeed, great variability was observed in the results obtained from the same sample, highlighting the impossibility of relying on the product in terms of the amounts of the active ingredient.

These aspects could adversely affect the effectiveness of the final product. In fact, the use of standardized and titrated extracts is essential for the treatment to be effective and reproducible over time. Standardize means “make uniform”. The use of standardized extracts, which guarantee a constant and repeatable content of active ingredients in each production batch, allows ensuring the reproducibility of the nutraceutical’s health action. Given the normal tendency to the variability of natural products as a consequence of different factors (plant origin, cultivation conditions, climate, etc.), the standardization process must first concern the raw material. The selection in the field of uniform plant populations based on the content of functional substances, therefore, represents the first fundamental step in the process of standardization of botanical drugs and all products derived from it. The subsequent transformation process, which concentrates and confers the desired characteristics to the extract, must then guarantee, through the use of codified methods (GMP) and conducted in parallel with analytical laboratory controls, a finished product always with the same chemical (title in active ingredients) and physical (density, appearance, consistency, solubility) characteristics. It is possible to ensure the same qualitative and quantitative active molecules constantly only with the use of standard extracts. The use of standardized and titrated plant extracts has made it possible to significantly reduce the variability of the composition of the extract physiologically due to the plant (moisture content, plant origin, method and time of harvest), the extraction (extraction method, type of solvent, solvent concentration) and production processes (batch size, extraction speed). The quality of a nutraceutical is, therefore, a condition “*sine qua non”* for its efficacy and safety. However, the quality must necessarily be defined and controlled by objective values that rely on validated criteria and not on subjective and somewhat imaginative considerations. In other words, the quality of a dietary supplement based on mushroom or botanical extracts cannot be defined if the raw materials, formulation strategies and production processes are not clearly known. In this regard, an increasingly close and fruitful dialogue between the scientific community and regulatory authorities is desirable to protect the health of the consumer and control a market with strong legislative limits.

## 5. Conclusions

The medicinal mushroom market offers an arsenal of products proposed for the prevention of numerous diseases or risk factors. Although these products are considered safe for their “traditional use”, the analyses conducted by the authors suggest inconsistencies from a different point of view: the diversity of the mushrooms declared on the label and the real content in beta-glucans, as well as the presence of contaminants in concentrations higher than those required by law. Greater controls relating to the qualitative and quantitative analysis of nutraceutical extracts, in addition to close dialogues between the scientific community and regulatory authorities, are urgent in order to regulate a market with strong legislative limits and protect the health of the consumer.

## Figures and Tables

**Table 1:** List of mushroom-based supplements commercial products analyzed in the survey.

Sample	Batch	Active Ingredient (as Indicated on the Label)
1	A	*Lentinula edodes* (Shiitake)
2		*Ganoderma lucidum* (Reishi)
3		*Agaricus blazei*
4	B	*Lentinula edodes* (Shiitake)
5		*Ganoderma lucidum* (Reishi)
6		*Agaricus blazei*
7	C	*Lentinula edodes* (Shiitake)
8		*Ganoderma lucidum* (Reishi)
9		*Agaricus blazei*
10	D	*Ganoderma lucidum* (Reishi)
11		*Agaricus blazei*
12		*Lentinula edodes* (Shiitake)
13	E	*Agaricus blazei*
14		*Ganoderma lucidum* (Reishi)
15		*Lentinula edodes* (Shiitake)
16	F	*Ganoderma lucidum* (Reishi)
17		*Agaricus blazei*
18		*Lentinula edodes* (Shiitake)
19	G	*Lentinula edodes* (Shiitake)

**Table 2:** Molecular identification, based on the homology of the ITS sequence of the fungal bioactive ingredients with sequences deposited in GenBank (https://www.ncbi.nlm.nih.gov/genbank/, accessed on 1 December 2022). Abbreviation: active ingredient, AI.

Sample	Batch	AI (as Indicated on the Label)	Identification	Homology (%)	Reference Accession Number (GenBank)
1	A	*Lentinula edodes* (Shiitake)	*Lentinula edodes*	99	*KY494570.1*
2		*Ganoderma lucidum (Reishi)*	*Ganoderma resinaceum*	100	*MK415305.1*
3		*Agaricus blazei*	*Ganoderma resinaceum*	98	*MK554775.1*
4	B	*Lentinula edodes (Shiitake)*	*Lentinula edodes*	98	*KY494570.1*
5		*Ganoderma lucidum (Reishi)*	*Ganoderma lucidum*	88	*JQ520187.1*
6		*Agaricus blazei*	*Degraded DNA*		
7	C	*Lentinula edodes (Shiitake)*	*Grifola frondosa*	99	*AY049123.1*
8		*Ganoderma lucidum (Reishi)*	*Grifola frondosa*	100	*FJ766486.1*
9		*Agaricus blazei*	*Grifola frondosa*	99	*MN646229.1*
10	D	*Ganoderma lucidum (Reishi)*	*Ganoderma lucidum*	100	*MW554083.1*
11		*Agaricus blazei*	*Agaricus subrufescens*	99	*AJ244543.1*
12		*Lentinula edodes (Shiitake)*	*Lentinula edodes*	99	*MW375037.1*
13	E	*Agaricus blazei*	*More than one amplicon (yeasts)*		
14		*Ganoderma lucidum* (Reishi)	*Ganoderma sichuanense*	93	*JF915398.1*
15		*Lentinula edodes (Shiitake)*	*More than one amplicon (yeasts)*		
16	F	*Ganoderma lucidum (Reishi)*	*Ganoderma resinaceum*	99	*MG706242.1*
17		*Agaricus blazei*	*Cordyceps militaris*	*92*	*AY725790.1*
18		*Lentinula edodes (Shiitake)*	*Lentinula edodes*	*99*	*MN622787.1*
19	G	*Lentinula edodes (Shiitake)*	*Degraded DNA*		

**Table 3:** Concentration of ergosterol (ERG, µg g^−1^ DW) for sample. Values are given as samples mean ± SD (standard deviation, n = 3).

Sample	Batch	ERG (µg g^−1^)	
		Mean	SD
1	A	452.85	134.48
2		561.60	40.54
3		151.93	47.78
4	B	34.38	18.56
5		332.58	55.51
6		43.60	13.16
7	C	129.91	54.87
8		31.32	5.31
9		40.75	10.06
10	D	15.87	3.80
11		20.19	1.90
12		3.45	0.77
13	E	1.79	0.11
14		132.20	16.36
15		21.53	5.17
16	F	44.58	1.11
17		70.29	2.91
18		52.14	1.09
19	G	8.54	0.55

**Table 4:** Analysis of variance of ergosterol content in a panel of 19 mushroom-based supplements between the mean values determined on 3 different capsules.

Source of Variation	Degrees of Freedom	Mean Square	F	*p*-Value	F Crit
Between Groups	18	76,985.130	48.463	<0.001	1.882
Within Groups	38	1588.544			
Total	56				

**Table 5:** Concentration of Aflatoxins (AFTs, expressed as the sum of B1, B2, G1 and G2 AFTs, µg kg^−1^ DW) for sample. Values are given as samples mean ± SD (standard deviation, n = 3); if concentration was below the method detection limit, values were reported as n.d. = not detected. * Values higher than that allowed by Commission Regulation (EC) No 1881/2006 of 19 December 2006, setting maximum levels for certain contaminants in foodstuffs (http://data.europa.eu/eli/reg/2006/1881/2022-01-01, accessed on 1 December 2022).

Sample	Batch	AFTs (µg kg^−1^)	
		Mean	SD
1	A	0.12	0.01
2		2.68	0.30
3		1.21	0.01
4	B	0.17	0.01
5		3.16	0.48
6		n.d.	
7	C	2.62	0.06
8		1.72	0.29
9		4.99 *	0.24
10	D	n.d.	
11		n.d.	
12		n.d.	
13	E	n.d.	
14		n.d.	
15		n.d.	
16	F	n.d.	
17		n.d.	
18		n.d.	
19	G	n.d.	

**Table 6:** Analysis of variance of total aflatoxins content in a panel of 19 mushroom-based supplements between the mean values determined on 3 different capsules.

Source of Variation	Degrees of Freedom	Mean Square	F	*p*-Value	F Crit
Between Groups	18	6.327	11.305	<0.001	1.882
Within Groups	38	0.560			
Total	56				

**Table 7:** Concentration of metals content (iron, Fe; arsenic, As; cadmium, Cd; mercury, Hg and lead, Pb expressed as µg g^−1^ DW) for sample. Values are given as samples mean ± SD (standard deviation, n = 3). Values higher than that allowed by Commission Regulation (EC) No 1881/2006 of 19 December 2006, setting maximum levels for certain contaminants in foodstuffs (http://data.europa.eu/eli/reg/2006/1881/2022-01-01, accessed on 1 December 2022).

Sample	Batch	Fe (µg g^−1^)	As (µg g^−1^)	Cd (µg g^−1^)	Hg (µg g^−1^)	Pb (µg g^−1^)
1	A	62.359 ± 16.989	<0.05	<0.05	<0.01	<0.05
2		19.664 ± 2.545	<0.05	<0.05	<0.01	<0.05
3		8.238 ± 1.501	<0.05	<0.05	<0.01	<0.05
4	B	26.865 ± 3.983	<0.05	<0.05	<0.01	<0.05
5		23.636 ± 3.505	<0.05	<0.05	<0.01	<0.05
6		104.018 ± 15.424	<0.05	<0.05	0.046 ± 0.005	<0.05
7	C	8.860 ± 1.314	<0.05	0.072 ± 0.012	<0.01	0.131 ± 0.011
8		62.295 ± 9.237	<0.05	<0.05	<0.01	<0.05
9		110.803 ± 16.429	<0.05	0.302 ± 0.020	0.026 ± 0.002	0.071 ± 0.025
10	D	2.912 ± 0.432	<0.05	<0.05	<0.01	<0.05
11		6.688 ± 0.992	<0.05	<0.05	<0.01	<0.05
12		2.041 ± 0.303	<0.05	<0.05	<0.01	<0.05
13	E	7.511 ± 1.114	<0.05	<0.05	<0.01	<0.05
14		4.720 ± 0.700	<0.05	0.086 ± 0.006	<0.01	0.059 ± 0.011
15		6.116 ± 0.907	<0.05	0.238 ± 0.010	0.035 ± 0.010	0.123 ± 0.013
16	F	15.026 ± 2.228	<0.05	<0.05	<0.01	<0.05
17		27.286 ± 4.046	<0.05	<0.05	<0.01	<0.05
18		13.341 ± 1.978	<0.05	<0.05	<0.01	<0.05
19	G	55.001 ± 1.928	<0.05	<0.05	<0.01	<0.05

**Table 8:** Analysis of variance of iron, cadmium, mercury and lead contents in a panel of 19 mushroom-based supplements between the mean values determined on 3 different capsules.

Metal	Source of Variation	Degrees of Freedom	Mean Square	F	*p*-Value	F Crit
Iron	Between Groups	18	3347.24	66.725	<0.001	1.882
	Within Groups	38	50.16			
	Total	56				
Cadmium	Between Groups	18	22.464	627.678	<0.001	1.882
	Within Groups	38	0.036			
	Total	56				
Mercury	Between Groups	18	0.683	100.5607	<0.001	1.882
	Within Groups	38	0.006			
	Total	56				
Lead	Between Groups	18	5.509	101.025	<0.001	1.882
	Within Groups	38	0.055			
	Total	56				

**Table 9:** Total glucan content determined in 19 mushroom-based supplements in single capsule (Cps 1 to 5) and on the mixture derived from 5 capsules (Mix of 5 cps) and expressed in g 100 g^−1^ DW.

Sample	Total Glucan Content (g 100g ^−1^ DW)								
	Cps 1	Cps 2	Cps 3	Cps 4	Cps 5	Min	Max	Mean	Mix of 5 Cps
1	36.70	38.39	35.79	27.57	28.92	27.57	38.39	33.47	26.23
2	30.47	30.78	29.62	32.83	24.18	24.18	32.83	29.58	29.12
3	42.12	57.84	46.17	32.08	26.81	26.81	57.84	41.01	30.41
4	28.02	28.13	24.22	30.22	23.76	23.76	30.22	26.87	27.83
5	74.56	78.16	34.92	50.25	39.75	34.92	78.16	55.53	33.05
6	40.49	44.26	41.92	37.19	40.26	37.19	44.26	40.82	38.53
7	31.83	34.24	31.91	39.79	31.42	31.42	39.79	33.84	36.51
8	30.47	39.78	31.03	25.67	19.15	19.15	39.78	29.22	21.37
9	37.64	32.16	30.69	47.68	31.66	30.69	47.68	35.97	33.05
10	41.33	40.17	37.86	33.06	28.26	28.26	41.33	36.14	31.42
11	28.64	29.80	26.83	23.21	22.35	22.35	29.80	26.16	23.75
12	53.23	52.91	46.41	35.98	31.37	31.37	53.23	43.98	35.35
13	56.82	48.81	57.43	60.05	53.22	48.81	60.05	55.27	50.29
14	56.50	49.28	55.76	57.66	51.61	49.28	57.66	54.16	55.55
15	50.47	48.92	46.61	47.15	56.54	46.61	56.54	49.94	60.34
16	49.73	44.03	41.18	41.86	40.30	40.30	49.73	43.42	47.49
17	24.81	24.68	22.58	25.31	24.36	22.58	25.31	24.35	25.08
18	40.47	43.87	36.75	44.71	40.14	36.75	44.71	41.19	39.82
19	36.08	34.34	31.84	35.64	34.60	31.84	36.08	34.50	35.84

**Table 10:** Analysis of variance of total glucan content in a panel of 19 mushroom-based supplements between the mean values determined on 5 different capsules and the mixture derived from 5 capsules.

Source of Variation	Degrees of Freedom	Mean Square	F	*p*-Value	F Crit
Between Groups	18	189.715	7.122	<0.001	2.182
Within Groups	19	26.638			
Total	37				

## Data Availability

The data presented in this study are available in the article.

## Abbreviations

AFTs	Aflatoxins
ERG	Ergosterol
GC-MS	Gas chromatography–mass spectrometry
GMP	Good Manufacturing Practice
ITS	Internal Transcribed Spacers
NCBI	National Center for Biotechnology Information
PCR	Polymerase Chain Reaction
TCM	Traditional Chinese Medicine
UHPLC	Ultra-High-Performance Liquid Chromatography
